# Beyond the Graft: Recurrence of Interstitial Lung Diseases Post Transplant

**DOI:** 10.3390/jcm14041093

**Published:** 2025-02-08

**Authors:** Prince Ntiamoah, Atul C. Mehta

**Affiliations:** 1 Department of Interventional Pulmonology, Advocate Aurora Health, Green Bay, WI 54311, USA; prince.ntiamoah@aah.org; 2 Department of Pulmonary Medicine, Respiratory Institute, Cleveland Clinic Foundation, Cleveland, OH 44195, USA

**Keywords:** interstitial lung disease, idiopathic pulmonary fibrosis, lung transplantation, connective tissue disease, bronchoscopy, primary disease recurrence, lymphangioleiomyomatosis, sarcoidosis, pulmonary Langerhans cell histiocytosis, hard metal lung disease

## Abstract

Interstitial lung diseases (ILDs) represent a heterogenous group of lung disorders marked by inflammation and/or fibrosis of the lung parenchyma, often leading to progressive shortness of breath and end-stage respiratory failure. In the U.S., ILDs affect approximately 650,000 individuals and cause approximately 25,000–30,000 deaths annually. Lung transplantation (LTx) offers definitive treatment for advanced ILD, with improved survival attributed to advancements in immunosuppression, organ preservation, surgical techniques, and postoperative care. However, disease recurrence in transplanted lungs remains a significant concern. Understanding the risk factors and mechanisms underlying recurrence is critical for refining recipient selection and improving outcomes. This review examines ILD recurrence post LTx and its implications for lung transplantation success.

## 1. Methodology for Study Selection

This narrative review was conducted to synthesize the current body of evidence on the recurrence of interstitial lung disease (ILD) after lung transplantation. A systematic approach was followed to ensure the comprehensiveness and accuracy of the review while adhering to established guidelines for narrative reviews. A comprehensive search of the literature was performed using the following electronic databases: PubMed, Scopus, Web of Science, and Embase. Keywords and Medical Subject Headings (MeSH) terms related to ILD recurrence after lung transplantation were employed, including but not limited to “interstitial lung disease”, “lung transplantation”, “recurrence”, “graft survival”, and “post-transplant outcomes”. Boolean operators (e.g., AND, OR) were utilized to combine terms and refine results. The search results were imported into reference management software to remove duplicates.

## 2. Introduction

Lung transplantation (LTx) has evolved over the past 40 years into a standard treatment for patients with end-stage lung diseases. Since the inception of the International Society for Heart and Lung Transplantation (ISHLT) registry, approximately 70,000 adult LTx procedures have been recorded, with a median survival of 6.2 years for recipients [1,2]. The true number of lung transplants is likely higher, as the ISHLT Registry is voluntary and does not capture all transplant centers worldwide.

To be eligible for LTx, patients must meet strict criteria, including a greater than 50% two-year mortality risk without transplantation, a more than 80% probability of surviving at least 90 days post-transplant, and an equally high likelihood of five-year survival with adequate graft function. Re-transplants make up around 5% of all lung transplants [1,3,4].

Progressive pulmonary fibrosis develops in about 30% to 40% of individuals with ILD, typically leading to respiratory failure and a median survival of 2.5 to 3.5 years without treatment [5,6]. Two anti-fibrotic drugs have shown promise in slowing disease progression and reducing the rate of forced vital capacity (FVC) decline in patients with confirmed or probable usual interstitial pneumonia (UIP) [7,8,9].

ILDs encompass a variety of diffuse parenchymal lung conditions that share common clinical, radiographic, and pathological features. Idiopathic pulmonary fibrosis (IPF), which is the most prevalent, also has the worst prognosis [1]. While the term “interstitial” points to the focus on the interstitium in ILD pathology, this term is somewhat misleading, as most ILDs also involve extensive alterations in alveolar and airway architecture.

Common causes of ILD include occupational and environmental exposures, such as organic or inorganic dust, drug-induced toxicity, and radiation-related lung injury. ILDs are also associated with connective tissue diseases (CTDs) [5,10]. Idiopathic causes are also extensive [11,12].

Treatment generally focuses on addressing the underlying causes; however, progressive cases often lead to fibrosis and necessitate lung transplantation.

ILD-related cases account for 21% of LTx worldwide, with IPF being the leading cause [13]. However, the recurrence of ILD post LTx remains a significant challenge to long-term success. Genetic factors, such as TSC and TTR mutations, and host immune responses have been increasingly recognized as contributing to post-transplant outcomes [14,15].

This review explores disease-specific recurrence patterns and highlights strategies to improve management and outcomes in LTx recipients.

## 3. Disease-Specific Recurrence Patterns

### 3.1. Interstitial Lung Diseases

In 2018, ILD accounted for 60% of all lung transplants performed in the United States, a substantial increase from 20.4% in 2006 [4,16]. Idiopathic pulmonary fibrosis (IPF), the most common ILD, carries the worst prognosis. According to the 2019 report by the International Society for Heart and Lung Transplantation, the median survival after lung transplantation for idiopathic interstitial pneumonias, including IPF, was 5.2 years, compared to 6.7 years for other ILDs and 6.2 years across all lung transplant recipients [3,4]. While bilateral LTx is generally favored, studies suggest it does not confer a survival advantage over SLT [17,18]. The primary goals of LTx are to extend survival and improve quality of life, but the recurrence of ILDs in transplanted lungs remains a significant concern. Factors such as age, sex, EGFR mutations, and the underlying disease play a role in recurrence risk, underscoring the importance of personalized post-transplant care [1].

The recurrence rates of ILDs following LTx vary depending on the specific subtype. DIP is the most frequently reported. King et al. described a case of DIP recurrence as early as one month post LTx, which resulted in patient death [19]. Verleden and Kotecha reported cases of recurrence at 12 and 14 months, respectively, with both patients recovering fully [20,21]. Atypical bacteria, viral, and fungal infections may have contributed to disease recurrence.

Bhatt et al. documented NSIP recurrence in a middle-aged female post bilateral LTx, with histology showing recipient-origin macrophages progressing to interstitial fibrosis as early as two months post transplant [22]. This highlights the role of host factors in recurrence. Similarly, Kern et al. reported a case of hypersensitivity pneumonitis recurring three years post LTx due to continued antigen exposure [23] Table 1.

There have been reported instances of recurrence of CTD-related ILDs following LTx. One such case involved a 15-year-old patient with polymyositis who underwent bilateral LTx but experienced disease recurrence. Postmortem examination revealed pulmonary fibrosis consistent with usual interstitial pneumonia [49]. Additionally, Scallan et al. described a unique case of fibrotic NSIP (NSIP-F) recurrence after bilateral LTx, which manifested as de novo antisynthetase syndrome (anti-SS) more than 2 years after transplantation. This case represents the first documented instance of such an occurrence [50]. The recurrence may be attributed to the development of tissue-specific autoantibodies, potentially triggered by an episode of acute rejection occurring one month post transplant, despite the absence of connective tissue disease in the donor [50,51]. More research is essential to identify the risk factors contributing to ILD recurrence post LTX and to refine preventive and management strategies.

### 3.2. Impact of Telomere Length on Lung Transplantation for Interstitial Lung Disease

Advancements in precision genomic medicine have greatly improved the ability to associate clinical phenotypes with abnormal cellular pathways and their underlying genetic mutations. Telomeres—repetitive nucleotide sequences at chromosome ends—serve to protect against chromosomal degradation [52,53]. Telomere dysfunction has been linked to idiopathic pulmonary fibrosis (IPF), impaired alloreactive immune responses, and poorer outcomes after lung transplantation. A heritable, age-adjusted short telomere (ST) length, defined as below the 10th percentile, has been strongly associated with IPF onset [14,53,54]. As many as one-third of individuals with familial IPF exhibit shortened telomeres or harbor mutations in telomere-related genes, while telomere-related mutations are found in approximately 10% of those with sporadic IPF [55]. Regardless of ILD subtype, individuals with short telomeres or known telomere-related mutations tend to experience faster disease progression and a reduced time to lung transplantation or death [55]. For example, transplant recipients with *TERC* mutations have been found to have a higher risk of bone marrow dysfunction [56].

Due to the connection between telomere-related mutations and ILD, along with the possibility of extrapulmonary organ dysfunction and immune system disturbances, there is increasing attention within the lung transplant community paid to refining the evaluation and the management of such patients. In some ILD programs, telomere length measurement has become routine; however, most U.S. transplant centers have yet to widely adopt this practice for evaluating lung transplant candidates with IPF [57]. Studies comparing transplant recipients with ST to those with normal telomere length have yielded mixed results regarding survival and graft function. While some studies report higher incidences of PGD, leukopenia, and reduced CLAD-free survival, others find no significant link between ST and outcomes such as PGD, ACR, time to CLAD, or long-term post-transplant survival [54,55,56,58,59]. These inconsistencies likely arise from variations in how ST is defined across studies. Findings from a single-center study by Newton et al. suggested that ILD recipients with telomeres below the 10th percentile experienced increased rates of severe primary graft dysfunction, a shorter time to developing CLAD, and reduced survival [56]. Additionally, various studies have shown mixed results on how donor telomere length affects lung transplant function [56,58]. Pathologic analysis of lung tissue from recipients with CLAD showed that their telomeres are shorter compared to age-matched controls (lungs from unused donors). However, it is still uncertain whether this shortened telomere length is due to a genetic predisposition from the donor’s telomere length or if the telomeres specifically shorten in the lung tissue of recipients as a result of CLAD [60].

Although lung transplantation mitigates the effects of telomere dysfunction in the lung parenchyma, systemic effects may still influence immune function. Lung transplant recipients with IPF and ST (IPF-LTRs) face an elevated risk of complications, including infections like cytomegalovirus and Epstein–Barr virus, post-transplant lymphoproliferative disease, and a higher prevalence of rare mutations in key telomere-related genes [53,57]. Given these risks, individuals with ILD being evaluated for lung transplantation should undergo telomere length screening if they have a personal history of early graying (before the age of 30), strong family history of pulmonary fibrosis, or signs of extrapulmonary organ dysfunction linked to short telomere syndrome [14,55]. For transplant recipients with telomere-related mutations, immunosuppression regimens should be carefully tailored based on bone marrow reserve. If feasible, T-cell-depleting agents like antithymocyte globulin should be avoided, since these have been linked to telomere shortening and reduced telomerase activity in real transplant patients [61].

Despite these findings, the effects of shortened telomeres on post-transplant outcomes remain inconclusive. Gaining a deeper insight into how telomere-related mutations influence ILD in the context of lung transplantation will aid in optimizing post-transplant management and refine individual care strategies for these patients, and possibly prevent retransplantation.

### 3.3. Sarcoidosis

Sarcoidosis is a multisystem disorder characterized by the formation of noncaseating granulomas and an exaggerated T-cell-mediated immune response [62,63,64]. While spontaneous remission occurs in a significant number of cases (approximately 80% for stage I, 60% for stage II, and 30% for stage III), 10–15% of patients progress to end-stage lung disease, with respiratory failure being the leading cause of mortality (1–6%) [62,65,66,67,68].

Lung transplantation (LTx) is a recognized treatment option for advanced sarcoidosis, particularly in cases with pulmonary hypertension, chronic respiratory failure, or resting hypoxemia [31,69]. Between 1995 and 2018, 1540 lung transplants (2.4% of all procedures) were performed for sarcoidosis, with 5-year survival rates ranging from 47% to 69% [26,31,70].

Among interstitial lung diseases, sarcoidosis is the most likely to recur following LTx, with reported recurrence rates between 14% and 47% [31]. Typically, granulomas reappear within 6–12 months post transplant, originate from the recipient’s immune cells, and rarely compromise graft function [71,72]. Through DNA analysis, Ionescu et al. confirmed that granulomas in the transplanted lungs are recipient derived [73]. The first case series documenting recurrent sarcoidosis appeared in 1993, describing recurrences occurring as early as two weeks and as late as two years post transplant [24,73,74]. Bilateral LTx may reduce the risk of recurrence compared to single LTx by offering increased functional reserve and mitigating infections linked to persistent bronchiectasis [28,31] Table 1.

Recurrent sarcoidosis often manifests with milder symptoms compared to the initial disease, likely due to the effects of immunosuppressive therapies. It can present as solitary or miliary nodules [24,25,26,28,29,30,31,72,74,75,76,77]. Because granulomas may appear in scattered or localized patterns, a negative biopsy does not definitely exclude recurrent sarcoidosis. Transplanted organs in recipients with pre-existing sarcoidosis are prone to developing sarcoid granulomas, while grafts from donors with known sarcoidosis rarely exhibit significant disease progression when transplanted into recipients without sarcoidosis. “Donor-acquired sarcoidosis” refers to cases where recipients presumed naïve develop the condition after receiving organs from donors not known to have active sarcoidosis [73].

Although the exact cause of sarcoidosis remains unclear, it is believed to result from a combination of genetic susceptibility and exposure to environmental or microbial triggers, such as *Mycobacterium tuberculosis* or *Propionibacterium acnes* [78]. Granulomas in transplanted lungs may reflect the infectious nature of the disease, as documented cases have shown transmission from donors to recipients [78,79,80]. A decline in recurrence rates since 2013 coincides with the increased use of mTOR inhibitors, which are thought to suppress granuloma formation [31,81,82]. Additionally, the switch from cyclosporine to tacrolimus in the early 2010s may have contributed to improved outcomes, although this hypothesis requires further investigation [31].

While granulomas in transplanted lungs are a potential marker of disease recurrence, they rarely impair graft function or survival and are generally managed with corticosteroids [72,73]. In cases where recurrence of sarcoidosis leads to respiratory failure, retransplantation may be considered, but this decision should account for the risks and benefits of the procedure. It is essential to rule out extrapulmonary involvement before proceeding. Further research is required to validate the long-term effectiveness of evolving therapies in preventing recurrence.

### 3.4. Lymphangioleiomyomatosis (LAM)

LAM is a rare, slowly progressive systemic disease characterized by cystic lung destruction and chylous fluid accumulation due to abnormal smooth muscle-like cell infiltration [83]. Primarily affecting women, LAM is associated either with tuberous sclerosis complex (TSC-LAM) or with sporadic TSC2 mutations (sporadic LAM) [36,84,85]. Common symptoms include progressive dyspnea and recurrent Pneumothoraces, which contribute significantly to morbidity and often necessitate repeated medical interventions [84,86,87].

The disease course of LAM is highly variable, ranging from mild symptoms to severe respiratory failure. In some cases, median survival exceeds 20 years [88]. Histological analysis reveals that LAM cells express both melanoma-related and smooth muscle antigens, which aid in diagnosis [87,89]. Management primarily focuses on symptom control complication management. Sirolimus, an MTOR1 inhibitor, has been shown to stabilize lung function, reduce serum VEGF-D levels, and improve quality of life, although its discontinuation often leads to disease progression [87]. Everolimus, another mTORC1 inhibitor, has also demonstrated efficacy in multiple trials [90,91].

For patients with end-stage LAM, lung transplantation (LTx) remains the definitive treatment, offering better post-transplant survival compared to other advanced lung diseases [1,86,92]. The use of MTOR inhibitors has reduced the need for lung transplantation in some LAM patients; however, LTX is still indicated for those with severely reduced lung function (<30%), significant exertional dyspnea (NYHA class III or IV), or resting hypoxemia [1]. The first reported LTx for LAM was a combined heart–lung transplant in 1984 [93]. By 2019, 582 LTx procedures for LAM had been performed worldwide [3]. Survival after LTx is generally favorable, with one study reporting a median survival of 12 years post transplant, including survival rates of 94% at one year, 73% at five years, and 55% at ten years [94]. The recurrence of LAM following lung transplant is rare, with rates of 6–7% and only 23 reported cases [32,33,34,35,36,38,76,77,87,91,95,96,97,98,99,100,101], Table 1. Recurrence can occur as early as two months post transplant, but survival outcomes remain excellent.

Genetic studies suggest that LAM recurrence in the allograft results from metastatic LAM cells, potentially driven by immunosuppression and genetic predisposition [36,84,87,88,102]. Effective management requires careful balancing of the benefits of mTOR inhibitors, such as reduced recurrence risk, against potential complications like bronchial dehiscence, particularly during the immediate postoperative period [103,104]. Premature discontinuation of sirolimus prior to transplantation has been associated with rapid declines in lung function [104,105,106]. Since bronchial dehiscence typically occurs 4–6 weeks post transplantation, some centers opt to continue sirolimus up to the time of surgery or switch to everolimus, a shorter-acting mTORC1 inhibitor, to maintain pre-transplant stability while minimizing post-surgical complications [105,106].

Retransplantation in LAM is rare, with only five cases documented, including two due to graft failure and one resulting from bronchiolitis obliterans syndrome. Notably, no cases of retransplantation have been specifically attributed to LAM recurrence [86,107,108]. It remains unclear whether mTOR inhibitors or hormonal therapies could prevent or delay recurrence in the transplanted lung [38,109].

The rarity of LAM presents challenges for conducting large, randomized trials, limiting the evidence base for optimal management. Nonetheless, sirolimus is recommended as a primary therapy for LAM, either alone or in combination with calcineurin inhibitors (CNI) [38]. Alternative regimens may include second-line antiproliferative agents, such as everolimus, or substitutions like mycophenolate or azathioprine. The long-term benefits of lifelong mTOR inhibitor therapy remain an area for further research.

### 3.5. Pulmonary Langerhans Cell Histiocytosis (PLCH)

PLCH is a rare cystic lung disease strongly associated with smoking, primarily affecting young adults and potentially leading to pulmonary hypertension and respiratory failure. This disease arises from a myeloid dendritic cell disorder and accounts for 3–5% of adult diffuse parenchymal lung diseases, with over 90% of cases linked to cigarette smoking [10,110]. The typical age of onset is between 20 and 40 years, with an equal incidence among males and females, although women tend to present at older ages [10]. There are no known occupational or geographic predispositions; however, nearly all affected individuals have a history of current or prior smoking [111,112].

Langerhans cells, normally present in small quantities in the dermis, reticuloendothelial system, lungs, and pleura, play a central role in PLCH pathogenesis. Smoking is believed to drive the accumulation of CD1a-positive dendritic cells that harbor oncogenic mutations, such as BRAF, V600E, and MAPK2K1 [113]. These mutations activate the MAPK pathway, a key mechanism underlying disease progression. Diagnosis is supported by the detection of CD1a- and CD207-positive cells in the bronchoalveolar lavage (BAL) samples [114,115].

In asymptomatic or mild cases, smoking cessation and regular monitoring are often sufficient. For progressive disease, systemic glucocorticoids provide limited benefits, while refractory cases may require treatment with cytotoxic agents such as cladribine or cytarabine, which necessitate careful monitoring for cytopenias and infection risks [116].

Lung transplantation (LTx) is a therapeutic option for patients with advanced, progressive PLCH and constitutes approximately 0.4% of adult transplants [117]. Post-transplant survival rates are 77% at one year and 54% at ten years [118]. Recurrence of PLCH in the transplanted lung is rare, occurring in roughly 10% of cases, typically within 5–60 months post transplant [39,40,41,42,119]. Risk factors for recurrence include resumption of smoking and preoperative extrapulmonary involvement [41].

The first documented case of PLCH recurrence was reported in a 32-year-old nonsmoker who underwent bilateral LTx and experienced recurrence two years later [40]. Subsequent reports, such as those by Etienne et al., describe recurrence following single LTx in patients who resumed smoking after surgery [42] Table 1. Recurrence may be driven by extrapulmonary factors or the local proliferation of abnormal Langerhans cells, which may exhibit neoplastic-like behavior [39]. The tendency of PLCH to recur in similar pulmonary distributions before and after transplantation remains poorly understood.

A multicenter study of 39 patients found that PLCH recurrence occurred in 10% of cases, with no significant impact on survival [41,118]. Extrapulmonary involvement, however, was a significant risk factor for recurrence. Management typically includes smoking cessation and corticosteroids, with pulse steroid therapy often providing symptom relief and resolution of pulmonary infiltrates [119]. Retransplantation for PCLH is rare and has been performed for bronchiolitis obliterans syndrome rather than for disease recurrence.

While recurrence of PLCH in the transplanted lung does not appear to significantly affect overall survival, preoperative extrapulmonary involvement remains a critical risk factor. Even in candidates for retransplantation, the potential for recurrence of the primary disease must be considered.

### 3.6. Pulmonary Alveolar Proteinosis

Pulmonary alveolar proteinosis (PAP) is a condition marked by the accumulation of lipoproteinaceous material within the alveoli, resulting from impaired surfactant clearance by alveolar macrophages [120,121]. It is classified into three types, genetic, secondary, and autoimmune, with the autoimmune variant being the most prevalent [122]. In autoimmune PAP, antibodies targeting granulocyte–macrophage colony-stimulating factor (GM-CSF) disrupt macrophage function, leading to defective surfactant processing [123]. The clinical presentation varies, ranging from asymptomatic cases to symptoms such as dyspnea, cough, fatigue, chest pain, fever, and hemoptysis. Diagnosis is supported by the identification of periodic acid–Schiff-positive eosinophilic granules within foamy macrophages [121].

The primary treatment for PAP is whole-lung lavage, which effectively clears accumulated surfactant in most patients. However, some individuals develop pulmonary fibrosis over time. In refractory cases, supplemental GM-CSF, rituximab, or plasmapheresis may be used [120,122,124]. For patients with end-stage disease, lung transplantation is a viable option, though disease recurrence after transplantation has been reported [124]. There have been three documented cases of recurrence, one associated with autoimmune PAP and two linked to genetic causes, occurring at 3-, 16-, and 26-months post-transplant [46,47,48]. Recurrence in genetic cases is thought to result from defective precursor macrophages originating from the bone marrow, raising the possibility that bone marrow transplantation could serve as an adjunct therapy to LTx in select patients.

Interestingly, PAP has also been identified in lung allografts of patients who underwent transplantation for unrelated conditions, including idiopathic Eisenmenger’s syndrome, pulmonary fibrosis, and pulmonary hypertension [125,126,127]. Additionally, a case was reported in a patient who developed acute myeloid leukemia five years after LTx and subsequently developed PAP following chemotherapy [128]. These patients tested negative for GM-CSF autoantibodies, suggesting that secondary PAP in transplant recipients may be triggered by alveolar injury due to ischemia, infection, or immunosuppressive therapy (Table 1). Despite the presence of these potential mechanisms in many LTx recipients, only a small subset develops PAP, highlighting the need for further research to identify additional contributing factors.

### 3.7. Hard Metal Exposure

Hard metal lung disease (HMLD) arises from prolonged occupational exposure to cobalt and tungsten carbide. Its defining pathological feature is giant cell interstitial pneumonia (GIP), which is considered pathognomonic for the disease but has also been reported in cases without confirmed metal exposure [45,129]. Unlike many other occupational lung diseases, the development of HMLD does not depend on the cumulative level of exposure [119,130]. Histologically, GIP is characterized by the presence of multinucleated giant cells exhibiting emperipolesis, a phenomenon where neutrophils and lymphocytes are engulfed within the giant cells [131].

The primary approach to managing HMLD is the cessation of exposure to hard metals, which can lead to recovery in non-fibrotic cases. However, once fibrosis develops, the lung damage is generally irreversible. While corticosteroids and immunosuppressive therapies have been used in treatment, their effectiveness has not been definitely established [132,133].

For patients with end-stage or progressive disease, LTx is a viable option. However, recurrence of the disease after transplantation has been reported, even in the absence of subsequent hard metal exposure [43,44]. Frost et al. described a case of single LTx with disease recurrence after two years, where autopsy findings revealed typical GIP changes in the allograft without evidence of inorganic particles [44]. Similarly, Tarabachi et al. documented recurrent lung injury characterized by multinucleated cells in the transplanted lung following single LTx [43]. Another notable case reported persistent GIP across native lungs, transplanted lungs, and re-transplanted lungs over an eight-year period in a patient without prior hard metal exposure (Table 1). This case suggests a potential autoimmune mechanism underlying disease recurrence [45]. Further research is needed to clarify the mechanisms driving recurrence in HMLD and to develop targeted strategies for post-transplant management and prevention of disease relapse.

## 4. Conclusions

The recurrence of interstitial lung diseases following transplantation is influenced by multiple factors, including recipient immune responses, persistent antigen exposure, donor–recipient immune interactions, and genetic predispositions such as short telomere syndromes. Understanding the pathophysiology and individual risk factors for recurrence has the potential to refine and guide personalized management strategies. Disease recurrence should be carefully evaluated as a potential cause of new-onset allograft dysfunction and must be excluded. Incorporating genetic screening into pre-transplant evaluations, particularly for high-risk patients with known telomere-related mutations, will be critical for optimizing long-term outcomes and advancing the field of lung transplantation.

### Future Directions

Further research is needed to elucidate the mechanisms of disease recurrence after lung transplantation, particularly the role of genetic (short telomere) and immune factors and immune responses. Additionally, improving post-transplant management strategies, including optimizing immunosuppressive regimens and exploring novel therapies for refractory cases, is essential to enhance graft survival and patient outcomes.

## Figures and Tables

**Table 1:** Features of recurrence of various ILDs following lung transplantation.

Reference	Primary Disease	Number of Patients with Recurrence	Type of Transplant	Patient Age (in Years), Mean	Post-Transplant Immunosuppressive Medications	Post-Transplant Complications	Outcomes
Martel S et al. [24]	Sarcoidosis	One	Single LTx	25	Patient was started on steroids, AZA and cyclosporine	Acute rejection occurred within the first year of LTx	There was primary disease recurrence 1 year 10 months post transplant
Nunley DR et al. [25]	Sarcoidosis	Five	Single LTx	44	Patients were on AZA, steroids, cyclosporin, or tacrolimus		The earliest primary disease recurrence was noted at 3 weeks. Four patients died from refractory acute rejection and opportunistic infections
Walker S et al. [26]	Sarcoidosis	Three	Single LTx		Steroids, AZA, and cyclosporine		Granulomas were found at 5-, 6- and 56 months post transplant. There was 56% survival at 5 years
Carre P et al. [27]	Sarcoidosis	One	Single	25	Steroids, AZA, and cyclosporine	Acute rejection	
Bjørtuft et al. [28]	Sarcoidosis	One	Single LTx	46		Acute rejection, CMV	Interestingly, recurrence occurred 26 weeks post transplant. After re-transplant, there was recurrence again at 46 weeks
Milman N et al. [29]	Sarcoidosis	Three	Single LTx	51	Steroids, AZA, and cyclosporine	Bronchiolitis obliterans	Recurrence occurred one to 6 months post transplant
Martinez et al. [30]	Sarcoidosis	One	Bilateral LTx	40			Disease recurred 13 months post transplant
Le Pavec et al. [31]	Sarcoidosis	Eleven	Bilateral LTx	52		Most of the patients developed CLAD	Recurrence occurred without 24 months. Three of the patients underwent re-transplant
Nine JS et al. [32]	LAM	One	Single lung transplant	45	Steroids, tacrolimus, and inhaled cyclosporine	Bronchiolitis Obliterans	Patient died from disseminated fungal infection. At autopsy, LAM recurrence was confirmed—3 years post transplant
Bittmann et al. [33]	LAM	One	Single lung transplant	34		Acute rejection	Patient died 2 years post transplant. Disease recurrence was confirmed on autopsy
O’Brien et al. [34]	LAM	One	Single LTx	42	Steroids, AZA, and cyclosporine	Acute rejection	Patient died from cholecystectomy complications. Recurrence was detected on autopsy—2 years post transplant
Sugimoto et al. [35]	LAM	One	Bilateral LTx	23	Steroids, AZA, and tacrolimus		Patient had worsening lung function 5 years post transplant. Imaging was suggestive of possible disease recurrence. Improved symptoms after sirolimus was started
Karbowniczek et al. [36]	LAM	One	Single LTx	44	Steroids, AZA, and cyclosporine		Patient died from invasive aspergillosis. LAM recurrence noted on autopsy—22 months post transplant
Taveira-Da Silva et al. [37]	LAM	One	Bilateral LTx	26		Fungal pneumonia	LAM recurred 36 months post transplant.
Zaki et al. [38]	LAM	One	Bilateral LTx	66	Steroids, mycophenolate, and tacrolimus	Multiple pneumonias from pseudomonas, chronic rejection	Patient developed disease recurrence on biopsies 9 years post transplant
Habib et al. [39]	PLCH	One	Bilateral LTx	28	Steroids, AZA, and cyclosporine	CMV pneumonia	Recurrence occurred 11 months post transplant
Gabbay et al. [40]	PLCH	One	Bilateral LTx	32	Steroids, AZA, and cyclosporine		Disease recurrence at 2 years post transplant
Dauriat et al. [41]	PLCH	Eight	Seven bilateral LTx One single LTx	31			The average time to recurrence was 28 months, with the earliest being 5 months post transplant. Three of these patients resumed smoking prior to recurrence. Four of the eight patients died
Etienne et al. [42]	PLCH	Two	Single LTx	26	Steroids, AZA, and cyclosporine	CMV pneumonia	Recurrence occurred 12 months post transplant
Tarabichi et al. [43]	Hard metal disease	One	Single LTx	45		Acute rejection	Patient had relapse and died after 2.4 years post transplant
Frost et al. [44]	Hard metal disease	One	Single LTx				Patient had recurrence after 2 years post transplant. Died
Ntiamoah et al. [45]	Hard metal disease	One	Bilateral LTx	40	Steroids, tacrolimus, and mycophenolate	Grade 3 PGD	Recurrence in native, transplanted, and retransplanted lung. Patient still alive 11 years post initial transplant
Takaki et al. [46]	Pulmonary Alveolar Proteinosis	One	Bilateral LTx	36			Disease recurred 16 months post transplant. Died
Santamaria et al. [47]	Pulmonary Alveolar Proteinosis	One	Bilateral LTx	3		EBV pneumonia	Recurrence occurred after 18 months. Patient died 26 months post transplant
Parker et al. [48]	Pulmonary Alveolar Proteinosis	One	Bilateral LTx	41		Bronchiolitis obliterans	Disease recurred 3 years post transplant
Kern et al. [23]	Hypersensitivity pneumonitis	One	Bilateral LTx	49			Primary disease recurrence after 3 years
Arboleda et al. [49]	Polymyositis-ILD	One	Bilateral LTx	15			Disease recurred after 9 months. Died.
Bhatt et al. [22]	Nonspecific interstitial pneumonia	One	Bilateral LTx	42		Grade 3 PGD	Primary disease recurrence several months post transplant
Kotecha et al. [20]	Desquamative interstitial pneumonia	One	Bilateral LTx	59		CMV pneumonia with AMR	Primary disease recurred 1 year 2 months post transplant
Scallan et al. [50]	Fibrotic Nonspecific interstitial pneumonia	One	Bilateral LTx	52		Developed Grade A2 rejection	Primary disease recurred 2.5 years post transplant
King et al. [19]	Desquamative interstitial pneumonia	One	Single LTx	52		Opportunistic infections (Nocardia, CMV)	Primary disease recurred just 4 weeks post transplant. Died.
Verleden et al. [21]	Desquamative interstitial pneumonia	One	Single LTx	51		PJP pneumonia, Acute rejection	Primary disease recurred 12 months post transplant.

AZA: azathioprine, CMV: cytomegalovirus, EBV Epstein–Barr virus, LAM: lymphangioleiomyomatosis, LTx: lung transplant, PGD: primary graft dysfunction, PLCH: pulmonary Langerhans cell histiocytosis, PJP: pneumocystis pneumonia
